# Protocol for prophylaxis of venous thromboembolism in varicose vein surgery of the lower limbs

**DOI:** 10.1590/0100-6991e-20223326-en

**Published:** 2022-08-17

**Authors:** MARCOS ARÊAS MARQUES, STÊNIO KARLOS ALVIM FIORELLI, BERNARDO CUNHA SENRA BARROS, ALCIDES JOSÉ ARAÚJO RIBEIRO, ARNO VON RISTOW, ROSSANO KEPLER ALVIM FIORELLI

**Affiliations:** 1 - Universidade Federal do Estado do Rio de Janeiro, Departamento de Cirurgia Geral e Especializada - Rio de Janeiro - RJ - Brasil; 2 - Universidade do Estado do Rio de Janeiro, Unidade Docente Assistencial de Angiologia - Rio de Janeiro - RJ - Brasil; 3 - Hospital de Base do Distrito Federal, Cirurgia Vascular - Brasília - DF - Brasil; 4 - Academia Nacional de Medicina - Rio de Janeiro - RJ - Brasil

**Keywords:** Venous Thromboembolism, Pulmonary Embolism, Patient Safety, Prevention & Control, Protocols, Tromboembolismo Venoso, Embolia Pulmonar, Segurança do Paciente, Prevenção e Controle, Protocolos

## Abstract

Pulmonary embolism is the most feared complication of venous thromboembolism (VTE) and the third leading cause of cardiovascular mortality in the world, after acute myocardial infarction and stroke. The risk of VTE is virtually universal in hospitalized patients, especially those with reduced mobility. Although variable in incidence between clinical and surgical patients, up to 66.6% of events related to hospitalizations can occur after discharge, with this risk remaining for up to 90 days. Despite all the investment made in VTE prophylaxis in recent decades, there is still no consensus or specific guidelines for its prevention in patients undergoing conventional surgery for varicose veins of lower limbs. The adoption of a validated risk assessment model for VTE prophylaxis, based on the current literature, may help in the implementation and standardization of VTE prophylaxis in conventional lower limb varicose vein surgery, in addition to this benefit, it may lead to a reduction in the length of hospital stay and the number of readmissions.

## RESUMO

O tromboembolismo pulmonar é a complicação mais temida do tromboembolismo venoso (TEV) e a terceira causa de mortalidade cardiovascular no mundo, atrás apenas do infarto agudo do miocárdio e do acidente vascular cerebral. O risco de TEV é praticamente universal nos pacientes hospitalizados, especialmente naqueles com redução da mobilidade. Embora variável em incidência entre os pacientes clínicos e cirúrgicos, até 66,6% dos eventos relacionados às internações, podem ocorrer após a alta, permanecendo este risco por até 90 dias. Apesar de todo investimento feito na profilaxia do TEV nas últimas décadas, ainda não existem consensos ou diretrizes específicos para a sua prevenção em pacientes submetidos à cirurgia convencional de varizes dos membros inferiores. A adoção de um modelo de avaliação de risco validado para a profilaxia do TEV, embasado na literatura vigente, poderá ajudar na implementação e padronização da profilaxia do TEV na cirurgia convencional de varizes de membros inferiores, além deste benefício, poderá levar a diminuição do tempo de internação hospitalar e do número de reinternações.

## INTRODUCTION

Pulmonary thromboembolism (PTE) is the most feared complication of venous thromboembolism (VTE) and the third leading cause of cardiovascular mortality in the world, after acute myocardial infarction and stroke^1^. It is estimated that approximately 10 million new cases of VTE occur each year worldwide^1^, but this number may be underestimated due to oligosymptomatic patients or those with nonspecific signs and symptoms of both PTE and deep venous thrombosis (DVT).

The risk of VTE is almost universal in hospitalized patients, especially in those with reduced mobility^2^ ^,^ ^3^. Although variable in incidence between clinical and surgical patients^1^, up to 66.6% of VTE events related to hospitalizations can occur after discharge, the risk remaining for up to 90 days^4^ ^-^ ^6^. Despite all the investment made in VTE prophylaxis in the last decades, there is still no consensus or specific guidelines for its prevention in patients who will undergo conventional surgery for varicose veins of the lower limbs (LL)^4^.

It is estimated that up to 60% of PTE episodes occur during or after hospitalization for clinical or surgical reasons^5^ ^,^ ^6^, but the concept that hospital admission represents an independent risk factor for VTE, as a nosocomial complication for both clinical and surgical patients, is still not clearly established in the entire medical community^7^.

In Brazil, despite a 31% reduction in age-adjusted mortality due to PTE in the last 21 years, from 3.04 to 2.09 per 100,000 inhabitants^7^, there is still an important variation between the country’s regions, probably due to differences in the quality of the services offered, in the absence or presence of continuing education for medical and non-medical teams, in the access and availability of complementary diagnostic methods, and failures in the disease notification.

In addition, even already well-established recommendations in guidelines for their efficacy and safety, such as VTE prophylaxis in surgical patients, often face great resistance to systematic adoption, due to the lack of knowledge and familiarity of the medical team with the subject, overestimation of possible adverse effects and complications, personal resistance to changes in well-established routines, or simply forgetting the topic^7^.

The implementation of a new guideline in a medical practice can be a complex task. The literature shows that, although routinely used, isolated measures, such as continuing education or the simple distribution of a protocol containing these recommendations, have little impact on behavior change^7^ ^,^ ^8^. The adoption of multiple conducts, such as the implementation of an easily applicable algorithm, continuing medical education, distribution of educational material, alerts in electronic or physical medical records, and the use of applications on cell phones, which help physicians to routinely apply the recommendations in their patients, is more effective than the adoption of any of these measures alone, and this implementation depends not only on the participation of each individual, but on the involvement of the entire patient care team (doctors, nurses, physiotherapists, and pharmacists). Moreover, the support of the hospital’s administration body is essential for encouraging preventive measures and for evaluating and obtaining results from the proposed intervention^8^.

On the other hand, there are measures known to be effective and safe for VTE prophylaxis (pharmacological and/or mechanical), making it the main cause of preventable hospital death^8^. However, there are some barriers to the adoption of such measures in hospitals, one of which is the difficulty in establishing a VTE systematic risk assessment, both in clinical and surgical patients^6^ ^-^ ^8^.

Several studies in Brazilian hospitals have observed underutilization of VTE prophylaxis, reiterating data from the ENDORSE study^9^, according to which the average adequacy of VTE prophylaxis in the world is around 50% in high risk clinical and surgical patients.

The American College of Chest Physicians (ACCP) 2008 guidelines^1^ for the prevention and treatment of VTE has established that the adoption of a systematization and a guideline or algorithm VTE prophylaxis, in any patient profile, is the responsibility not only of the medical team, but also of the institution^7^ ^,^ ^8^.

The use of a validated risk assessment model (RAM) for VTE prophylaxis, based on current literature, may help the adoption and standardization of VTE prophylaxis in conventional LL varicose vein surgery that will be performed and thus reduce the morbidity and mortality of this procedure. In addition to this benefit, it may lead to a reduction in the length of hospital stay and the number of readmissions, directly benefiting the patient and the Public Unified Health System (SUS), by reducing surgical costs and increasing bed availability for other procedures.

Adopting a standard for VTE prophylaxis in hospitals is a formal recommendation from numerous national and international guidelines, medical societies, and governmental institutions to help ensure patient safety^6^ ^-^ ^8^. However, this requires institutional and multidisciplinary participation and continuing education to be successful.

### Incidence and consequence of VTE in lower limb varicose vein surgery

The real incidence of VTE in conventional LL varicose vein surgery remains unknown, ranging in the literature from 0.4 to 5.3%^10^. Although some experts argue that oligosymptomatic distal DVT or PTE events have low morbidity and mortality, the socioeconomic and psychological impact of a DVT or PTE diagnosis cannot be overlooked.

From a practical point of view, most patients with these diagnoses, regardless of location, magnitude, or symptoms, will receive anticoagulant treatment, which involves high costs, risk of bleeding, and the need for medical follow-up and regular examinations, for at least six months^4^ ^-^ ^7^. In addition, the diagnosis of DVT or PTE during hospitalization can increase hospital stay, blocking this hospital bed for a longer time and, therefore, causing the postponement of some other procedure scheduled for another patient, which can lead to an increase in the queue and waiting time in the SUS, in addition to burdening it even more. And even if the VTE occurs outside the hospital environment, after discharge it can result in the patient being readmitted, which would end up causing the same types of problems mentioned above.

Another very important point to be considered is that patients who undergo surgery for LL varicose veins are often still economically productive, and the emergence of VTE, a potentially preventable complication, can lead to absenteeism from work, increased social security costs, and decreased family income.

### Risk Assessment Model (RAM)

VTE prophylaxis is a key component in the protection and safety of patients who will undergo conventional surgery for varicose veins of the lower limbs, and its effectiveness is related, among other things, to the identification of patients who are huger risk of developing it^4^ ^,^ ^11^ ^,^ ^12^ ^,^ ^14^.

Identifying which patients are at high risk for the development of postoperative VTE can be difficult in many cases, but there are several RAM that are useful and validated tools in the pre-procedure, admission, and hospital discharge approach, such as the Caprini score, which can be adopted for this purpose^4^ ^,^ ^11^ ^,^ ^12^.

### Caprini score (CS)

CS is an RAM validated in a large retrospective study with a sample of general, vascular, and urological surgery patients and used in practice to assess the risk of VTE in surgical patients, without distinction^12^ ^,^ ^15^.

CS is useful for individual VTE risk stratification and choice of the most appropriate conduct for pharmacological and/or mechanical prophylaxis. Moreover, it is a dynamic tool that can be used in the reassessment of the patient as many times as necessary, since the changes in their clinical status can cause changes in the score and, therefore, in the adopted conduct^12^ ^,^ ^14^.

CS (Table 1) is based on several clinical characteristics, with different scores varying between one and five points each, and classifies the surgical patient in general, including vascular patients, in four risk categories: very low risk (0 points), low risk (1-2 points), moderate risk (3-4 points), and high risk (≥5 points), with an estimated risk of developing VTE, when pharmacological prophylaxis and/or mechanics measures are not adopted, of <0.5%, 1.5%, 3.0%, and 6.0%, respectively^12^ ^,^ ^15^.

**Table 1:** Caprini Score: VTE risk assessment model for surgical patients.

1 point	2 points	3 points	5 points
41-60 years old	61-74 years old	>75 years old	Stroke (<1 month)
Minor surgery	Arthroscopy	VTE history	Elective arthroplasty
BMI* >25kg/m^2^	Neoplasia	Family history of VTE	Fracture of hip, pelvis, or leg
Lower limb edema	Major surgery (>45 minutes)	Leiden’s Factor V	Acute spinal trauma (<1 month)
Varicose veins	Laparoscopy (>45 minutes)	Prothrombin 20210 mutation	
History of recurrent or unexplained miscarriage	Restriction to bed (>72 hours)	Lupus anticoagulant	
OCC** or HRT#	Plaster immobilization	Anticardiolipin antibody	
1 point	2 points	3 points	5 points
Sepsis (<1 month)	Central venous access	Elevated serum homocysteine	
Severe lung disease, including pneumonia (<1 month)		Heparin-induced thrombocytopenia	
Abnormal lung function		Other acquired or hereditary thrombophilia	
Acute myocardial infarction			
Congestive heart failure (<1 month)			
Inflammatory bowel disease			
Clinical patient confined to bed			
Pregnancy or puerperium			

*BMI: body mass index; **Oral contraceptive; #Hormone replacement therapy. Adapted from Gould MK, Garcia DA, Wren SM, et al. Prevention of VTE in nonorthopedic surgical patients: antithrombotic therapy and prevention of thrombosis, 9th ed: ACCP evidence-based clinical practice guideline. Chest. 2012;141(2 Suppl):e227S-e277S.

Patients classified as moderate (3-4 points) or high risk (≥5 points) by CS have a reduced incidence of VTE when undergoing pharmacoprophylaxis, the risk of major bleeding not outweighing the benefits. Therefore, this conduct ends up offering a satisfactory efficacy and safety ratio^4^ ^,^ ^12^ ^,^ ^15^.

Patients classified as very low (0 points) or low risk (1-2 points) have a lower risk of developing VTE and thus should not receive pharmacoprophylaxis, as the risk of major bleeding may outweigh the proposed benefits^4^ ^,^ ^12^ ^,^ ^15^.

### Assessment of bleeding risk

Few studies or guidelines describe or classify risk factors for bleeding related to pharmacoprophylaxis in general. The ninth edition of the ACCP Antithrombotic Therapy and VTE Prophylaxis Guideline suggests a list of risk factors for major bleeding in vascular surgery based on risk factors for general, abdominal, and pelvic surgery (Table 2) as a basis for evaluating the patient bleeding risk profile^15^.

**Table 2:** Risk factors for major bleeding in surgical patients.

General risk factors	Active bleeding
	Prior major bleeding
	Known untreated coagulopathy
	Severe liver or kidney dysfunction
	Thrombocytopenia
	Acute cerebrovascular accident
	Lumbar puncture, epidural, or spinal anesthesia 4 hours before or 12 hours after
	Concomitant use of anticoagulant, thrombolytic or antiplatelet
Risk factors inherent to the procedure	Abdominal surgery: male, hemoglobin <13g/dL, neoplasia, and complex surgery
	Pancreatoduodenectomy: sepsis, sentinel bleeding, and leakage
	Liver resection: number of segments, extrahepatic resection, primary liver cancer, anemia, and thrombocytopenia
	Cardiac surgery: use of ASA or clopidogrel up to 3 days before the procedure, BMI* >25kg/m^2^, emergency surgery, advanced age, >5 bypasses, chronic renal failure, and long surgical time
	Thoracic surgery: pneumonectomy or extensive resection
Procedures for which bleeding consequences can be potentially dangerous	Craniotomy, spinal surgery, spinal trauma, and reconstructive surgery involving vascularized or non-vascularized free flaps

*BMI: body mass index. Adapted from Gould MK, Garcia DA, Wren SM, et al. Prevention of VTE in nonorthopedic surgical patients: antithrombotic therapy and prevention of thrombosis, 9th ed: ACCP evidence-based clinical practice guideline. Chest. 2012;141(2 Suppl):e227S-e277S.

### Current recommendations for VTE prophylaxis in lower limb varicose vein surgery

There is still a great deal of discussion about the best form of VTE prophylaxis for patients who will undergo conventional LL varicose vein surgery^4^ ^,^ ^10^ ^,^ ^16^ ^-^ ^18^. It is important to emphasize that this procedure is elective, with a usually short hospital stay, with no initial need for bed rest, using locoregional anesthesia, and having short duration^4^ ^,^ ^10^ ^,^ ^16^. Therefore, it usually has a low risk of VTE, despite the presence of varicose veins itself being considered an independent risk factor in CS (1 point)^4^.

The National Institute for Health and Care Excellence (NICE) guideline recommends that all patients should be mandatorily evaluated for the risk of VTE and bleeding at hospital admission and reassessed 24 hours after the procedure for readjustment of prophylaxis, if necessary. This guideline also recommends the adoption of pharmacoprophylaxis for patients classified as high risk for developing VTE who will undergo conventional surgery for LL varicose veins^13^.

The ninth ACCP guidelines for the prevention and treatment of VTE^15^ comprise the management of prophylaxis in different clinical and surgical situations, suggesting the use of a RAM to categorize the risk of patients who will undergo different types of surgeries, including vascular ones (venous or arterial) and, according to this risk, suggests the use of mechanical (intermittent pneumatic compression or elastocompression) and/or pharmacological methods (low molecular weight heparin - LMWH - or unfractionated heparin - UFH).

### Simplified suggestion of prophylactic measures for VTE in lower limb varicose vein surgery, according to CS

#### Patients with very low (0 points) or low (1-2 points) risk

Early ambulation and elastocompression.

No pharmacoprophylaxis.

#### Patients with moderate (3-4 points) or high (≥5 points) risk

Early ambulation.

Elastocompression.

Pharmacoprophylaxis with LMWH (preferred) or UFH, if there is no increased risk of bleeding.

#### Patients with moderate (3-4 points) or high (≥5 points) risk, with contraindication to pharmacoprophylaxis with UFH or LMWH

Early ambulation.

Elastocompression.

Pharmacoprophylaxis with fondaparinux.

#### Patients with moderate (3-4 points) or high (≥5 points) risk, with contraindication to pharmacoprophylaxis (Table 3)

**Table 3:** Contraindications to the use of pharmacoprophylaxis in lower limb varicose vein surgery.

Absolute	Relative
Patient already on anticoagulation for any reason	Recent intracranial or eye surgery
History of known allergy to any anticoagulant	Hemorrhagic diathesis
History of heparin-induced thrombocytopenia	Thrombocytopenia (<50,000)
Active bleeding	INR* >1.5
	Uncontrolled systemic arterial hypertension (>180 x 110mmHg)

* INR: international standardization ratio.

Early ambulation.

Elastocompression.

### Simplified dosage of VTE pharmacoprophylaxis for lower limb varicose vein surgery in moderate (3-4 points) or high (≥5 points) risk, according to CS

#### LMWH (preferred)

#### Enoxaparin

20mg (moderate risk) or 40mg (high risk), subcutaneously, once a day, for seven to ten days.

#### Dalteparin

2,500 UI (moderate risk) or 5,000 UI (high risk), subcutaneously, once a day, for seven to ten days.

#### UFH

5,000 UI, subcutaneously, every 8 hours (high risk) or every 12 hours (moderate risk), for seven to ten days.

### In cases of contraindication to UFH or LMWH

#### Fondaparinux

2.5mg, subcutaneously, once a day, for seven to ten days.

Pharmacoprophylaxis should be started within the first 24 hours after surgery. If anesthesia was by spinal blockade, prophylaxis management should be done according to Table 4.

**Table 4:** Management of pharmacoprophylaxis for neuraxial manipulation.

	Dose	Via	Dose interval for neuroaxis puncture (hours)	Interval of the last dose for catheter removal (hours)	Interval for administration after puncture or removal of the catheter (hours)
UFH*	5,000 UI, t.i.d. or b.i.d.	SC	4 to 6	4 to 6	1
LMWH**	20 or 40mg q.d.	SC	12	12	4

*UFH: unfractionated heparin; **LMWH: low molecular weight heparin.

## FINAL CONSIDERATIONS

Since, in daily practice, the medical team fails to systematically assess the risk of VTE and prescribe pharmacological or mechanical prophylaxis^19^ ^-21^, it is essential that the Vascular Surgery Team be responsible for promoting a protocol to conduct risk evaluation and guiding prophylaxis for patients considered to be at high risk. Some accreditation bodies ^21^ suggest that a RAM should be standardized, specific, individualized, linked to a menu of prophylaxis options (pharmacological and/or mechanical), and have a list of contraindications. The adoption of a VTE RAM is an ongoing process, with the involvement of the institution and several members of the health team, to make it part of the routine and thus improve management indicators.
